# Activation-Induced Conformational Changes of Dopamine D3 Receptor Promote the Formation of the Internal Water Channel

**DOI:** 10.1038/s41598-017-13155-z

**Published:** 2017-10-06

**Authors:** Wei-Hsiang Weng, Ya-Tzu Li, Hao-Jen Hsu

**Affiliations:** Department of Life Sciences, Tzu Chi University, Hualien, 97004 Taiwan

## Abstract

The atomic-level dopamine activation mechanism for transmitting extracellular ligand binding events through transmembrane helices to the cytoplasmic G protein remains unclear. In the present study, the complete dopamine D3 receptor (D3R), with a homology-modeled N-terminus, was constructed to dock different ligands to simulate conformational alterations in the receptor’s active and inactive forms during microsecond-timescale molecular dynamic simulations. In agonist-bound systems, the D3R N-terminus formed a “lid-like” structure and lay flat on the binding site opening, whereas in antagonist and inverse agonist-bound systems, the N-terminus exposed the binding cavity. Receptor activation was characterized using the different molecular switch residue distances, and G protein-binding site volumes. A continuous water pathway was observed only in the dopamine-G_αi_-bound system. In the inactive D3Rs, water entry was hindered by the hydrophobic layers. Finally, a complete activation mechanism of D3R was proposed. Upon agonist binding, the “lid-like” conformation of the N-terminus induces a series of molecular switches to increase the volume of the D3R cytoplasmic binding part for G protein association. Meanwhile, water enters the transmembrane region inducing molecular switches to assist in opening the hydrophobic layers to form a continuous water channel, which is crucial for maintaining a fully active conformation for signal transduction.

## Introduction

Dopamine (DA), a neurotransmitter abundant in the central nervous system (CNS), affects neuronal activity and regulates functions such as locomotor activity, memory, emotion, cognition, and reward. In the peripheral nervous system, DA facilitates the regulation of hormone secretion, vascular tone, renal function, and gastrointestinal motility^1^. DA functions by binding and activating its five cognate G protein-coupled receptor (GPCR) subtypes on cell surfaces. These DA receptors include two subfamilies, DA receptor D1 (D1R)- and D2R-like, which differ in their signal transduction, binding profile, and physiological effects^2^. D2R-like (D2R, D3R, and D4R) DA receptors favor the binding of G_αi_ upon activation, thereby inhibiting the activation of adenyl cyclase (AC), which reduces cyclic AMP (cAMP) production^2^. Dysregulation of these receptors has been implicated in various several pathological conditions such as Parkinson’s disease^3^, schizophrenia^4^, attention deficit hyperactivity disorder^5^, and drug addiction^6^. Furthermore, D2R and D3R have been signified as potential targets for drug development in ocular diseases such as glaucoma^7^.

The general understanding of GPCR activation initiated by agonist binding is that the receptors reorient the side chains of amino acids within the transmembrane (TM) domain, causing conformational changes on a global scale for G protein recruitment on the intracellular side^8^. In the absence of ligands, GPCRs are believed to be in a dynamical equilibrium between the inactive and active states. The binding of either a partial or full agonist is believed to increase the probability of the receptor assuming its active state^9^. Although antagonists and inverse agonists restrain the receptor in its inactive state, only antagonists can block the binding of both agonists and inverse agonists.

Furthermore, considerable information regarding the association of the N-termini of GPCRs with chemokine receptors is available. For example, the chemokine receptors CXCR4 and CCR5 are known to participate in human immunodeficiency virus type 1 (HIV-1) infection^10–12^. The N-terminal domains of the chemokine receptors CXCR1 and CXCR2 play crucial roles in their initial recognition of and binding with the chemokine interleukin-8 (IL-8), where it interacts with the N-terminal loop of IL-8^13,14^. Because they are involved in several functions, the importance of the GPCR N-terminal domain cannot be underestimated. In previous studies aimed at clarifying the complete activation mechanism of β_2_AR^15^ and the M2 muscarinic receptor^16^, the participation of the N-terminal domain was completely ignored.

In 2010, the structure of human D3R was resolved^17^, which was the first obtained DA receptor structure and remains the only one to date. However, only an inactive state (bound to the antagonist eticlopride) of D3R does not provide information regarding the conformational transition between the inactive and active states. In previous studies on β_2_AR, A_2A_AR and rhodopsin, continuous water pathways were found to correlate with receptor activation^18–22^. However, the connection between the activating conformational change of the receptor and the formation of the continuous water pathway remains ambiguous. Molecular simulations provide a means for elucidating the activation process of D3R. In the current study, a more complete D3R model was constructed by combining the crystallized structure with a homology-modeled N-terminus. Molecular docking was performed for D3R with agonists DA, and 7-OH-DPAT (7-hydroxy-*N,N*-dipropyl-2-aminotetralin), G_αi_ subunit, the antagonist eticlopride, and the inverse agonist haloperidol. The most preferable complexes were embedded in a lipid bilayer for further molecular dynamics (MD) simulations on a microsecond timescale to obtain a more complete picture of receptor activation. In the cases of antagonist eticlopride-bound D3Rs and inverse agonist haloperidol-bound D3Rs, the drugs remained in the orthosteric binding site during the 1.5 μs simulation time, and the two hydrophobic layers blocked the water molecules passing through the receptor. Our simulations suggest that when an agonist binds to D3R, the N-terminal conformational change induces the TM molecular switches to form an internal water channel and increases the volume of the cytoplasmic side, which is suitable for G protein binding. The continuous water channel of the activated D3R enables downstream signal transduction. The findings of this study elucidate how D3R assumes its active conformation, and could prove valuable in drug design for the treatment of CNS-related diseases.

## Results

**Figure 1:**
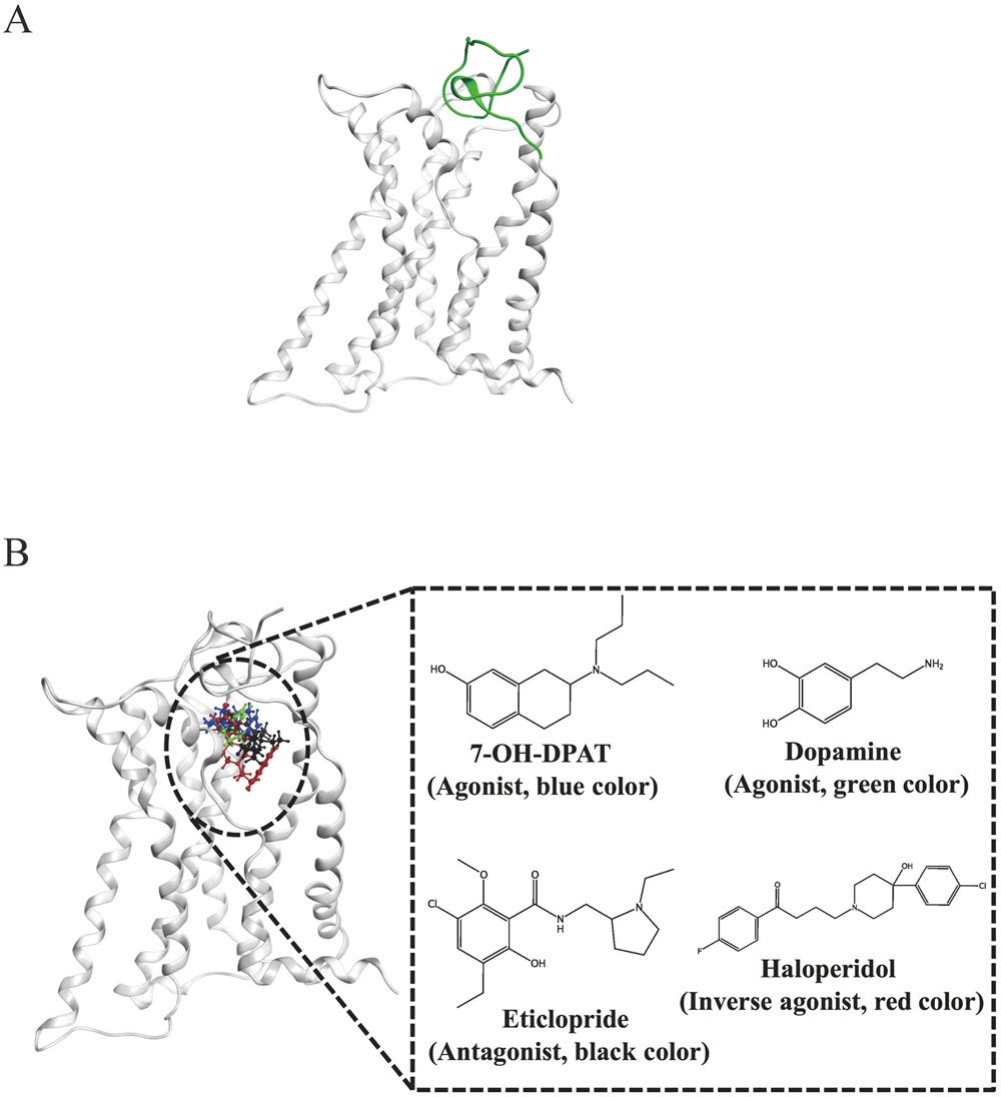
System setup of various D3R models. (**A**) The initial 200 ns average structure of apo D3R with the modeled N-terminus. (**B**) Preferable binding sites of different compounds docked to the modeled D3R.

To explore the role of the N-terminus in the activation mechanism of D3R, the N-terminal region (residues 1–31) of D3R was homology modeled to combine with the resolved D3R structure to develop a new D3R with the N-terminus. The modeled D3R structure was then embedded into a POPC lipid bilayer for equilibration. The average apo D3R structure for the first 200 ns MD simulations indicated that the N-terminus was a random coil partially occupying the N-terminal cavity (Fig. 1A).

### Dopamine D3 receptor docked with various small compounds

Molecular dockings of several compounds, such as 7-OH-DPAT (agonist), DA (endogenous agonist), eticlorpride (antagonist), and haloperidol (inverse agonist), to the equilibrated D3R structure were performed by using the *DOCK* module of MOE2015.10 software. To validate the molecular docking results, eticlopride was first redocked to D3R, and superposed with the crystal structure, the results of which indicated that the redocked position was similar to that observed in the crystal structure, which the portion of ECL2 (residues 182–185) contributes to the ligand binding (Supporting Figure S1A). In our docking results for various ligands, most of top 15 preferable poses of each ligand are in the binding pocket with lower binding free energy, whereas less poses are in the vestibule of receptor’s extracellular surface with higher binding free energy (Supporting Figure S1B–D and Tables S1–3). The preferable binding sites of all compounds docked to the modeled D3R were similar in binding cavity (Fig. 1B), showing that the cavity is suitable for various compounds (agonist, antagonist, or inverse agonist) binding. Our docking results were consistent with previous studies on other GPCR systems^17,19,20,22^, which the binding poses were in the binding cavity of GPCR.

### The N-terminal conformations of D3R with various drugs bound

**Figure 2:**
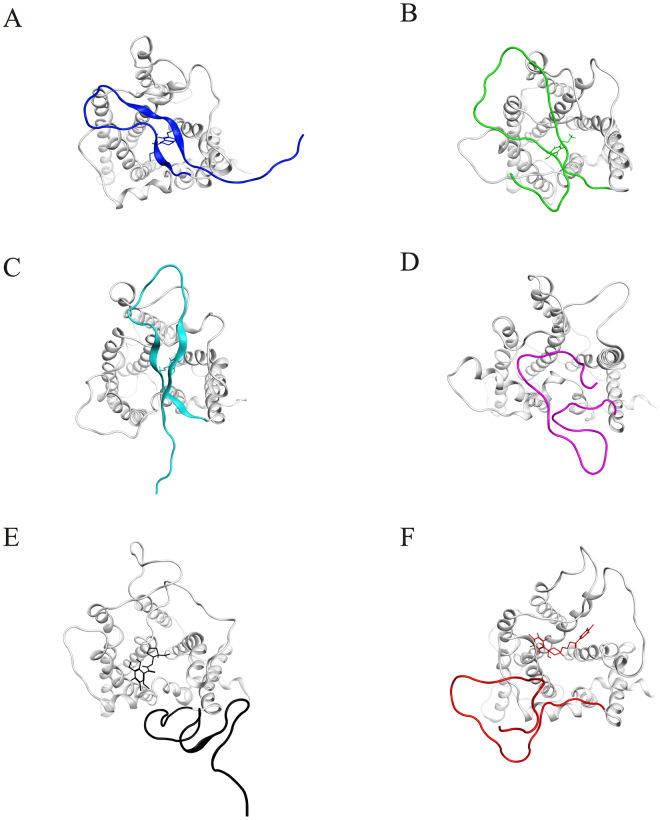
Different N-terminus conformations observed from the final 200 ns average structures of respective systems. (**A**) 7-OH-DPAT-bound D3R (**B**) DA-bound D3R (**C**) DA-G_αi_-bound D3R (**D**) apo D3R (**E**) Eticlopride-bound D3R and (**F**) Haloperidol-bound D3R. In 7-OH-DPAT-, DA- and DA-G_αi_-bound D3R systems, the N-terminus lay flat on the top of binding cavity, forming a “lid-like” conformation while in eticlopride- and haloperidol-bound systems, the N-terminus left the binding cavity exposed.

After molecular docking, D3Rs bound to various drugs were embedded into POPC lipid bilayers for more than 1.5 μs MD simulations. The average structures extracted from the final 200 ns simulations of each system indicated that in 7-OH-DPAT-, DA- and DA-G_αi_-bound systems, the N-terminus lay flat on top of the binding cavity, forming a “lid-like” conformation, whereas in the eticlopride and haloperidol systems, the N-terminus left the binding cavity exposed. In the apo D3R system, without any bound drug, the N-terminus lay on top, and was partially buried within the binding cavity (Fig. 2). To quantify the extent of “lid-like” conformation of the N-terminus, the contact area between the N-terminus and the TM region of D3R was calculated for various complex systems. The larger contact area is, the more extent of “lid-like” conformation is. The contact areas between the N-terminus and the TM region for 7-OH-DPAT-, DA-, DA-G_αi_-bound and apo D3R systems were calculated. These contact area range was from 7.7 to 10.5 nm^2^, which was consistent with the “lid-like” conformation of the N-terminus. The contact areas for eticlopride- and haloperidol-bound systems, by contrast, ranged from 3.5 to 5.8 nm^2^ (Supporting Figure S2). To exclude the possibility of stochasticity, the various drugs-bound complexes were repeated at least two times with different initial velocities to perform the first 200 ns MD simulations, indicating that the root-mean-square deviation (RMSD) values of the receptor backbone atoms of repeated simulations fluctuate near 0.45 nm (Supporting Figure S3A,B ,I, and J). The RMSD for each TM region is in the following, TM1~TM4 are around 1–2 Å, TM5~TM7 are around 1–3 Å. TM5~7 with larger RMSD than TM1~4 means that TM5~7 are related to the conformational changes induced molecular switches of D3R (Supporting Figure S3C–F). TM score analysis also showed that TM score values for dopamine- and haloperidol-bound D3Rs are all around 0.8, meaning that the structures after 200 ns MD simulations are quite similar to the initial models (Supporting Table S4). In addition, the larger RMSD may also be from the flexible N-terminus, ECL, and ICL regions. Furthermore, the structures extracted from the frame at 200 ns of the various drugs-bound D3Rs from repeated simulations were superposed, and similar results were observed at the N-terminal region when compared with the original simulations. The N-terminus of the DA- and 7-OH-DPAT-bound receptors formed “lid-like” structures over the binding cavity, and the N-terminus of the haloperidol- and eticlopride-bound receptors formed conformations that left the binding cavity exposed (Supporting Figure S3G,H ,K, and L).

### Molecular switches of D3R during MD simulations

GPCRs are often depicted as molecular machines that alternate between the inactive and active states through the conversion of molecular switches. In D3R, these switches communicate throughout the TM region to transmit signals from the extracellular ligand binding site to the cytoplasmic G protein binding site. To achieve a more reliable conclusion, another replica for each simulation system with different initial velocity was also performed to confirm our activation model (Supporting Figure S4).

### 3–7 lock switch

**Figure 3:**
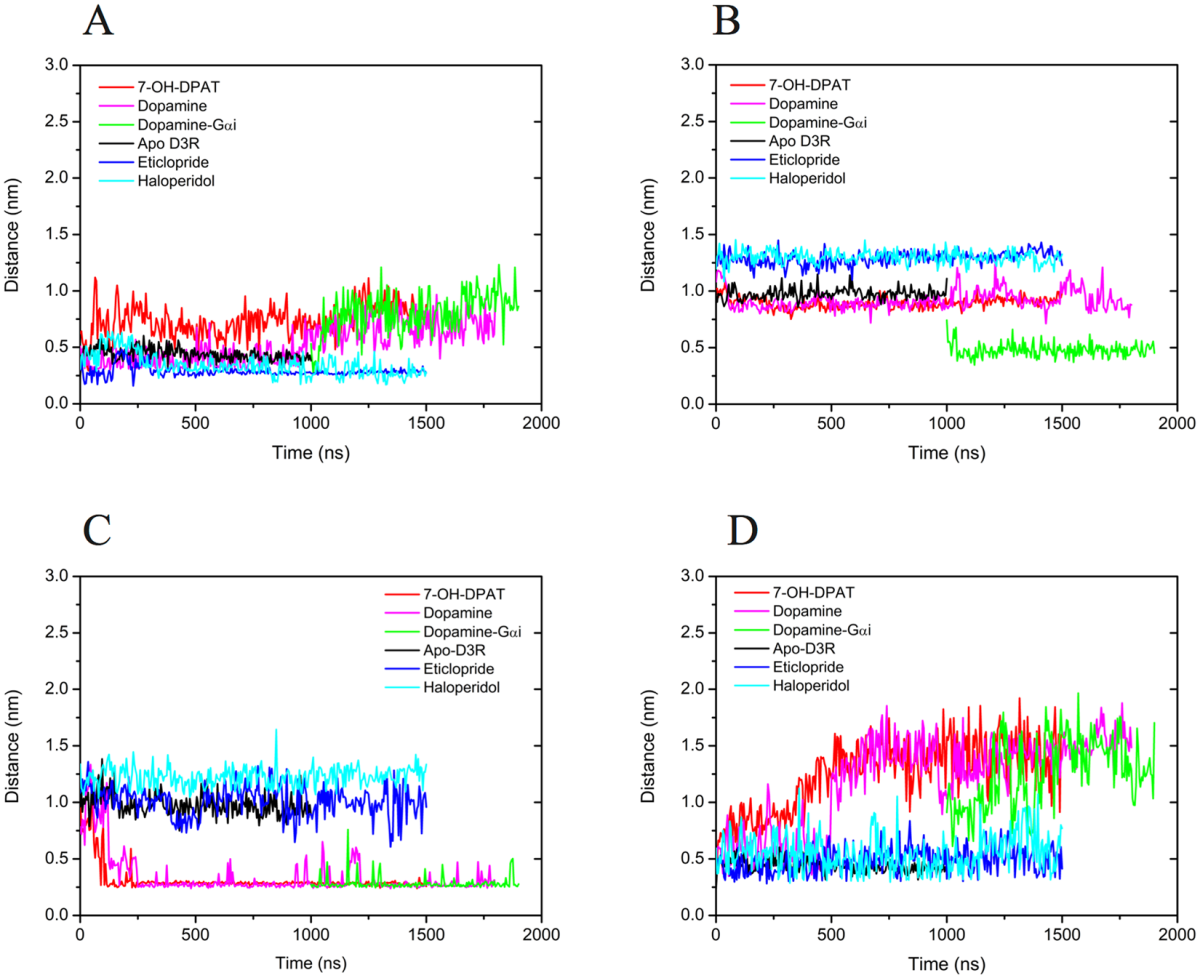
Measured distances between various molecular switches throughout the MD simulations. (**A**) 3–7 lock switch residues, the oxygen atoms of side chains of D110^3.32^ and Y373^7.43^ (**B**) Transmission switch, the center of masses (COMs) of W342^6.48^ and P200^5.50^ side chains (**C**) Tyrosine toggle switch, the oxygen atoms of side chains of Y208^5.58^ and Y383^7.53^ (**D**) Ionic lock switch, the nitrogen atom of R128^3.50^ and the oxygen atom of E324^6.30^. 7-OH-DPAT-, dopamine-, dopamine-G_αi_-, apo D3R, eticlopride-, and haloperidol-bound D3Rs are marked using red, pink, green, black, blue, and cyan lines, respectively.

The 3–7 lock switch was first observed in rhodopsin, where the salt bridge between E113^3.28^ and K296^7.43^ was confirmed to stabilize the receptor in its inactive conformation, and it was confirmed to be broken during activation^23^. The distances between the 3–7 lock switch residues, namely the oxygen atoms of side chains of D110^3.32^ and Y373^7.43^, were measured (Fig. 3A). Among the (agonist-bound) 7-OH-DPAT- and DA-bound D3Rs, the distances ranged from 0.5 to 1.0 nm after 1000 ns MD simulations, indicating that the salt bridge was broken to open the 3–7 lock. The distances measured for the (antagonist-bound) eticlopride- and (inverse agonist-bound) haloperidol-bound D3Rs were shorter and fluctuated near 0.35 nm after 500 ns MD simulations, indicating that the salt bridge was maintained; this was consistent with the previous resolved crystalized D3R structure, which has a distance between D110^3.32^ and Y373^7.43^ of 0.27 nm^17^.

### Transmission switch

The movements called “transmission switch” in the CWXP motif^24^ were measured between the center of masses (COMs) of the W342^6.48^ and P200^5.50^ side chains for various complex systems. The distances in the antagonist eticlopride- and inverse agonist haloperidol-bound D3Rs fluctuated near 1.3 nm, showing that TM6 rotated and W342^6.48^ moved away from P200^5.50^, similar to the rearrangements in TM5 and TM6 of β_2_AR and rhodopsin (Fig. 3B)^24^. As in the agonists 7-OH-DPAT-, DA-, and DA-G_αi_-bound D3Rs, the distances were shorter. They fluctuated near 0.85 nm for 7-OH-DPAT- and DA-bound systems and near 0.48 nm for the DA-G_αi_-bound system throughout the length of the simulation, in which W342^6.48^ moved toward P200^5.50^ (Fig. 3B). The transmission switch exhibited the relocation of conserved residues W342^6.48^ and F338^6.44^ toward Pro200^5.50^ during the activation of D3R, which can also be observed in several other GPCR systems such as rhodopsin receptor and A_2A_R^8,24^, consistent with present experiments.

### Tyrosine toggle switch

Continuous internal water pathways are critical mediators for the activation of GPCRs; however, hydrophobic barriers within the TM region hinder their formation. The hydrophobic barrier, consisting of six conserved residues between the TM helices TM2, TM3 and TM6, separates the water-mediated hydrogen bond network from the (D/E)RY motif and is related to the tyrosine toggle switch^25^. For apo D3Rs and eticlorpide- and haloperidol-bound D3Rs complex systems, the distances between the tyrosine toggle switch residues, namely the oxygen atoms of the side chains of Y208^5.58^ and Y383^7.53^, ranged from 0.95 to 1.30 nm, whereas the distances in the 7-OH-DPAT-, DA- and DA-G_αi_-bound D3Rs were measured to be approximately 0.3 nm after a simulation time of 350 ns (Fig. 3C). During the activation of D3R, the rotation of TM6 allows the hydrophobic barrier (V68^2.43^, L71^2.46^, L121^3.43^, I124^3.46^, M330^6.36^, and V334^6.40^) to open, and Y383^7.53^ of the NPXXY motif, together with Y208^5.58^, to be rearranged to fill the hydrophobic gap to extend the hydrogen bond network toward the DRY motif, resulting in the disruption of the ionic lock salt bridge, which also agrees with previous studies^8,25^.

### Ionic lock switch

The ionic lock was first identified as a strong intramolecular interaction between the residues E3.49/R3.50 of the conserved (D/E)RY motif in TM3 and the residues E6.30/T6.34 in TM6 for the inactive state of bovine rhodopsin^26^. The distances between the ionic lock residues, namely the nitrogen atom of R128^3.50^ and the oxygen atom of E324^6.30^, were measured to prove that the ionic lock was maintained in the (antagonist-bound) eticlorpide-bound and (inverse agonist-bound) haloperidol-bound D3R systems with distances fluctuating near 0.48 nm, which is a critical constraint that maintains the D3R system in the inactive form. However, in the agonists 7-OH-DPAT-, DA- and DA-G_αi_-bound D3R complex systems, the ionic lock was broken with increasing distances fluctuating near 1.45 nm after a simulation time of 800 ns (Fig. 3D). Furthermore, the D3R structures also exhibited an obvious kink in TM6, implying that the agonist binding triggers the ionic lock switch (R128^3.50^–E324^6.30^) to unlock the G protein binding site on the intracellular side of the receptor, leading to G protein activation. Our MD simulations were also comparable with those of several previous studies, such as antagonist-bound D3R^17^, inactive state of bovine rhodopsin structure^26^, and described activation mechanism of GPCRs^27^.

### Collective and correlated motions in D3R

**Figure 4:**
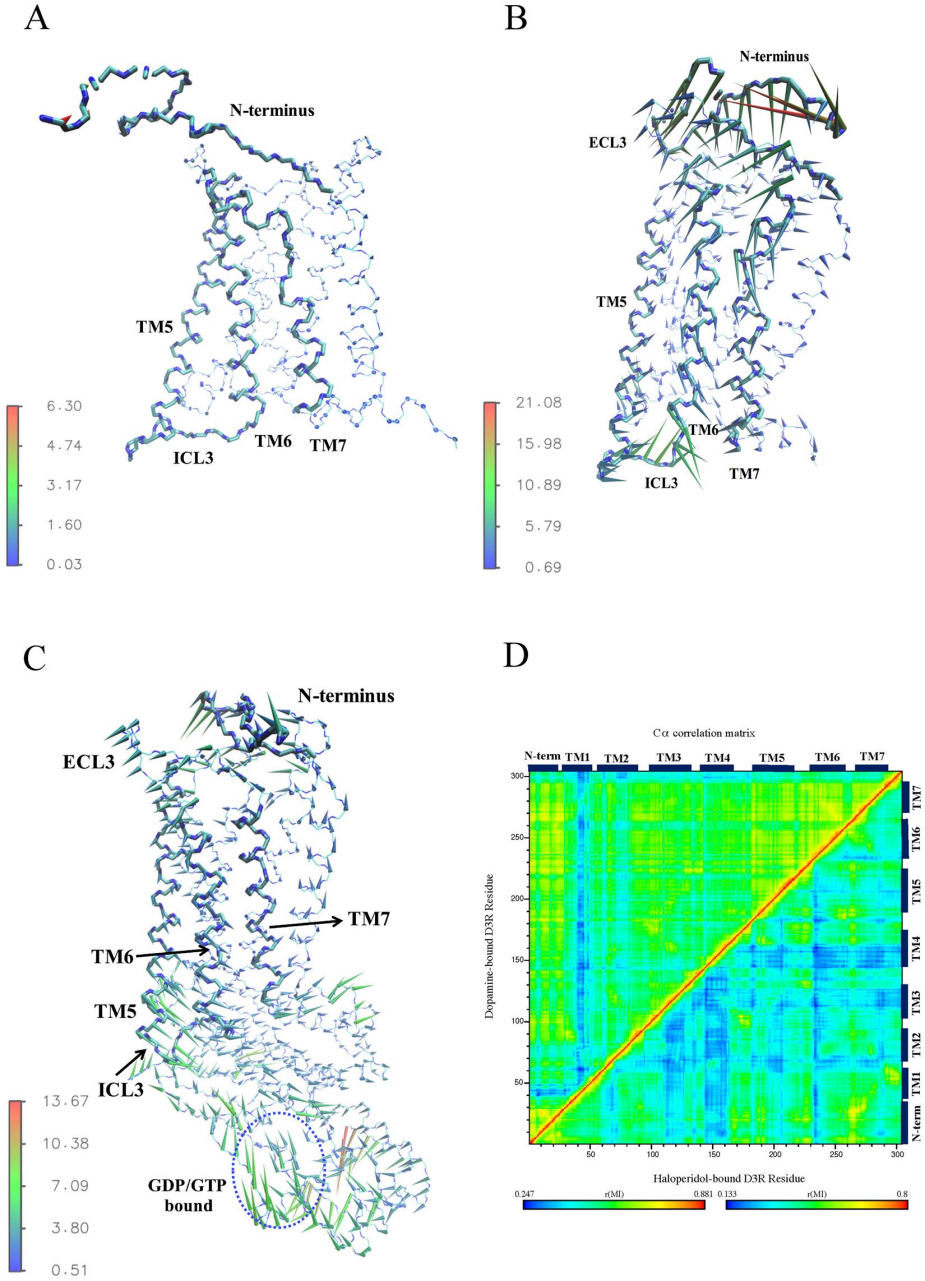
Dominant motions in various D3R systems using Principal Component Analysis and generalized cross correlation matrices. Porcupine plot of the first eigenvector in (**A**) Haloperidol (**B**) dopamine and (**C**) dopamine-G_αi_-bound D3R. Helices (TM5, 6, 7) and loops (N-terminus, ECL3, ICL3) along the first principal component are marked in light blue. Porcupine needles showed the direction and amplitude of harmonic motions. (**D**) generalized cross correlation analyses generated for dopamine (upper triangle) and haloperidol-bound (lower triangle) D3R C_α_ atoms. The strength of the computed correlation between two respective residues is color-coded (see color bars).

Principal component analysis was conducted to observe the collective motions of all C_α_ atoms of D3R to obtain sets of eigenvectors and eigenvalues. The collective modes of various complex D3R systems were dispersed over a total of 5454 eigenvectors, with the top eigenvector capturing approximately 25–40% of the total fluctuations, and the top 10 accumulating to approximately 70–80% of the total fluctuations listed in Table 1. Porcupine analyses were performed on the active states of the DA-, and DA-G_αi_-bound D3Rs and the inactive state of haloperidol-bound D3R (Fig. 4A–C). Porcupine needles indicate the direction and amplitude of harmonic motions. The haloperidol-bound receptor exhibited very small structural differences along the first principle component (Fig. 4A). By contrast, the DA- and DA-G_αi_-bound D3Rs exhibited relatively larger fluctuations, particularly those observed in TMs 5, 6, and 7, the N-terminus, extracellular loop (ECL) 3, and intracellular loop (ICL) 3, which directly interacts with G_αi_ (Fig. 4B and C). In addition, C_α_ atom correlated motions^28^ were calculated to indicate that in the haloperidol-bound receptor, most regions within the TM area were poorly correlated. By contrast, higher correlations of the DA-bound receptors were observed in the TM region, specifically, in most regions with the exception of TM1 (Fig. 4D). Collectively, these results imply that the activation of the DA-bound receptor may start from the N-terminus and transfer to the cytoplasmic side through correlated fluctuations with ECL3 and TMs 5, 6 and 7, thereby inducing a conformational change that allows intracellular G protein association.

**Table 1:** Eigenvalue percentage of top ten eigenvectors and of the first eigenvector in respective systems.

	Accumulated eigenvalues of top ten eigenvectors (%)	Eigenvalue of the first eigenvector (%)
7-OH-DPAT	74.57	30.73
DA	80.06	41.05
DA-G_αi_	71.30	24.86
Apo D3R	73.27	33.73
Eticlopride	74.22	41.44
Haloperidol	75.15	27.34

### Average volume of the G protein binding site in various complex D3R systems

**Figure 5:**
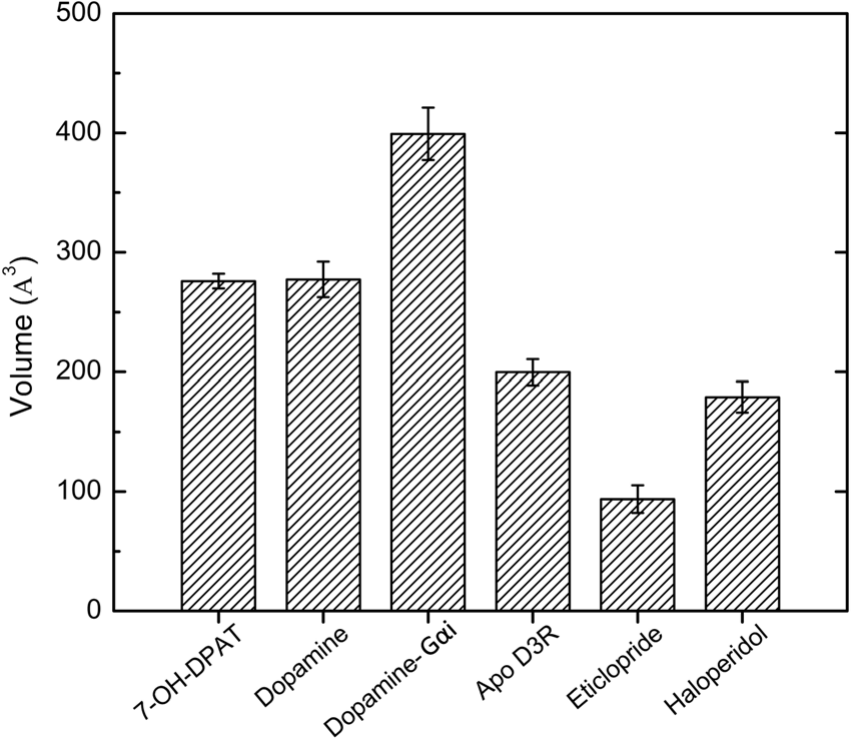
G protein-binding site volumes of final structures extracted from MD trajectories. The volumes were calculated with the PyMOL plugin KVFinder. The dopamine-G_αi_-bound receptor showed the largest volume, followed by the dopamine- and 7-OH-DPAT-bound receptors with similar volumes. Lower volumes were the apo D3R, haloperidol-, and eticlopride-bound D3 receptors.

Results from measuring the distances between known activation indicators of various GPCRs revealed that agonist-bound D3Rs showed signs of activation. Consistent with the notion that the conformational change that occurs on receptor activation allows G protein binding on the cytoplasmic side, the volumes of the G protein binding site of the final structures extracted from MD trajectories were calculated for different systems (Fig. 5). The DA-G_αi_-bound receptor exhibited the largest volume of approximately 400 Å^3^, followed by the DA- and 7-OH-DPAT-bound receptors with similar volumes (approximately 275 Å^3^). Lower volumes for the apo D3R, haloperidol-, and eticlopride-bound receptors were measured, ranging from 100 to 200 Å^3^, indicating that fewer conformational changes occurred on the cytoplasmic side of D3R, consistent with the porcupine analyses (Fig. 4A).

### Formation of the internal water channel in D3R

**Figure 6:**
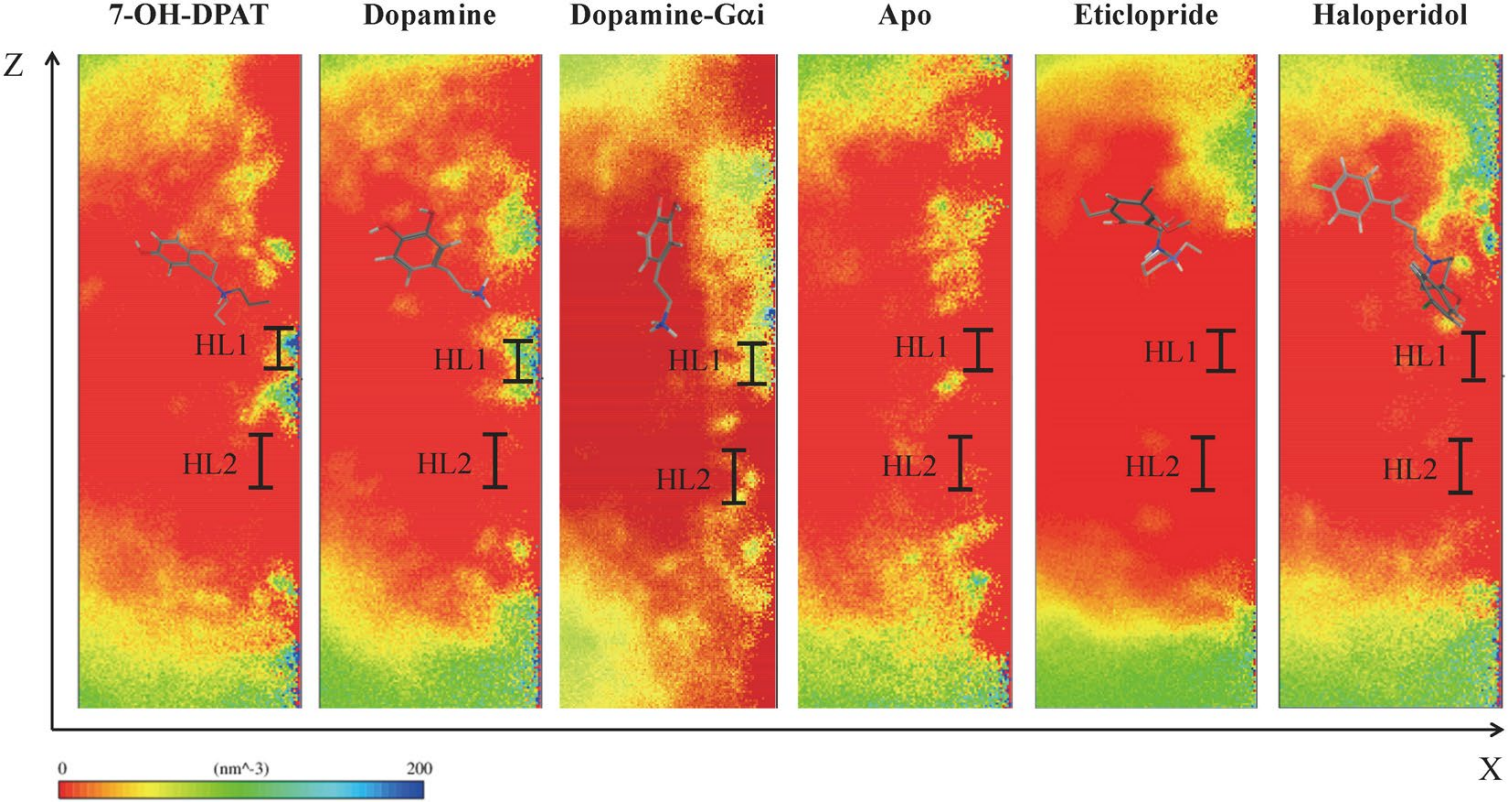
Water density maps of waters observed in the transmembrane region of respective systems. Water density distribution for various drugs bound systems were calculated, such as 7-OH-DPAT-, dopamine-, dopamine-G_αi_-, apo D3R, eticlopride-, and haloperidol-bound D3Rs (from left to right). HL1, and HL2 are the hydrophobic layers in the transmembrane region. The dopamine-G_αi_-bound receptor was most activated in comparison to other systems, which formed a continuous water pathway that extended from the extracellular side to the intracellular side.

The closer side view of the water density maps revealed that in 7-OH-DPAT- and DA-bound receptors, water molecules are grouped near the middle region of the TM domain and are hindered by a hydrophobic layer, HL2 (V68^2.43^, L71^2.46^, L121^3.43^, I124^3.46^, M330^6.36^, and V334^6.40^) (Fig. 6). Water molecules within the apo D3R, and the eticlopride- and haloperidol-bound receptors have a barely scattered entry from the extracellular side, and their movement is obstructed by the two hydrophobic layers, HL1 (A74^2.49^, V78^2.53^, F338^6.44^, W342^6.48^, V374^7.44^, and L378^7.48^) and HL2 (Fig. 6). Undoubtedly, the DA-G_αi_-bound receptor is more activated than are the other systems in this study. It forms a continuous water pathway extending from the extracellular side to the intracellular side (Fig. 6).

**Figure 7:**
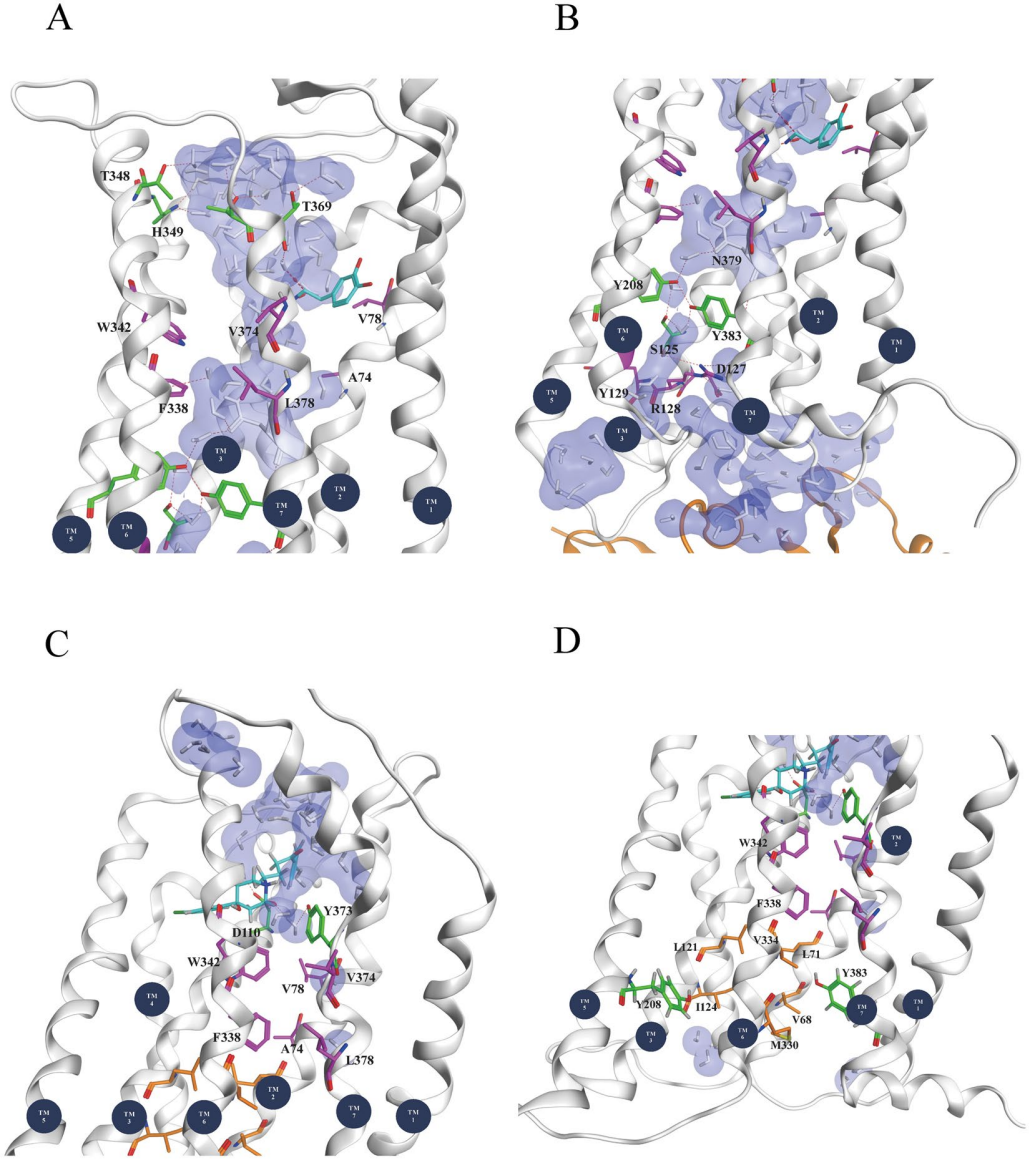
Internal waters pathway within the transmembrane region of two respective D3R systems. For dopamine-G_αi_-bound D3R system (**A**) upper half and (**B**) lower half regions. For haloperidol-bound D3R system (**C**) upper half and (**D**) lower half regions. Water molecules are drawn in both white sticks and blue molecular surface; drugs are marked in cyan sticks; the side chains of HL1 are marked in purple sticks (**A** and **C**), and the side chains of HL2 are marked in orange sticks (**D**).

In the last frame of the DA-G_αi_-bound receptor, water molecules within the TM region were mostly situated between TMs 3, 5, 6, and 7 (Fig. 7A). On the extracellular side of the receptor opening, the entry of water molecules into the TM domain was surrounded by T348, H349, T368 and T369. The nitrogen atom of the H349 imidazole side chain formed a hydrogen bond with a water molecule deep within the receptor. Moreover, the carboxyl oxygen atoms of T368 and T369 formed hydrogen bonds with other water molecules. The residues F338 and W342 in the hydrophobic layer HL1 rotated to approach TM5 to open the hydrophobic gap to enable the entry of water molecules. Further down the receptor, water molecules were forming hydrogen bonds with the side chain of N379 (Fig. 7B). The tyrosine toggle switch residues Y208 and Y383 disrupted the hydrophobic barrier HL2 to form a hydrogen bond network between the water molecules and surrounding residues such as S125, Y208, N379, and Y383. The hydrogen bond network extended toward the DRY motif (D127, R128, and Y129) near the cytoplasmic end and G protein.

In addition, in the last frame of the haloperidol-bound receptor, water molecules entering from the extracellular side were directed toward the region between TMs 2, 3, 6, and 7 (Fig. 7C). Haloperidol, D110, and Y373 formed multiple hydrogen bonds with these water molecules; however, the water molecules did not penetrate further into the hydrophobic layer HL1, which was surrounded by TMs 2, 6, and 7. Near the cytoplasmic side, no water molecules entered the TM region, which was similarly blocked by another hydrophobic layer HL2 (Fig. 7D).

## Discussion

### The role of N-terminus during D3R activation

Considerable information regarding the GPCR N-termini is related to chemokine receptors, whose N-termini play the role of recognizing approaching chemokines. The N-terminal domain of chemokine receptors CXCR1 and CXCR2 have been reported to interact with the N-terminal loop of the chemokine interleukin-8 (IL-8)^14,29,30^. IL-8 initially binds with the N-terminus of CXCR1 (site 1), followed by the orientation change to cause IL-8 to transfer and bind with the ECL residues (site 2) for downstream receptor activation^31–33^. In most *in silico* studies regarding the activation mechanism of GPCRs (e.g., in studies on β_2_AR^15^ and the M2 muscarinic receptor^16^), the involvement of the N-terminal domain has been completely ignored. However, in the current study, the N-terminus of D3R was observed to assume different conformations upon binding of different ligand types (Fig. 2). The results of simulations of the DA-, 7-OH-DPAT-, eticlopride- and haloperidol-bound systems with various initial velocities confirm that the different conformations of the N-terminus are not stochastic events because the results are reproducible (Supporting Figure S3). The N-terminal regions of D3Rs with the various ligands bound did not show large conformational changes after 150 ns MD simulations. The replicas of MD simulations for dopamine- and 7-OH-DPAT-bound D3R systems showed similar “lid-like” conformations whereas the replicas of MD simulations for haloperidol- and eticlopride-bound D3R systems showed the exposed binding cavity conformations. The repeated ligands, dopamine- and 7-OH-DPAT-bound D3Rs could represent the activation systems whereas eticlopride- and haloperidol-bound D3Rs represented inactivation systems. It is reasonable that the N-terminal regions of the replicas are not much similar due to the long flexible extracellular region, but they indeed formed “lid-like” and exposed binding cavity conformation related to activation mechanism. Agonist binding activates the receptors, whereas antagonist or inverse agonist binding inactivates the receptors. Here, based on our simulations, we propose that a “lid-like” conformation of the N-terminus appears to promote an agonist binding, thereby causing larger fluctuations in ECL3 and inducing breakage of the 3–7 lock switch salt bridge to allow the entry of water molecules. However, a conformation that exposes the binding cavity inhibits the binding of an antagonist or inverse agonist and maintains the 3–7 lock switch, which blocks the entry of water. In view of this phenomenon, additional investigations were conducted to elucidate the function of the N-terminal domain in small molecule systems. Extracting collective motions from DA-, and DA-G_αi_-bound D3Rs, larger fluctuations were observed starting from the N-terminus, continuing along TMs 5, 6, 7, ECL3, and then ending at ICL3. By contrast, haloperidol-bound D3R exhibited smaller fluctuations (Fig. 4A–C). Furthermore, the results of a cross correlation analysis of these two systems reveal that motions of the N-terminus of DA-bound D3R are highly correlated with those of TMs 2 to 7, whereas most regions of the haloperidol-bound D3R are poorly correlated (Fig. 4D). In summary, the different conformations of the D3R N-terminal domain also appear to play roles in receptor activation.

### Molecular switches correlate the activation mechanism and regulate the internal waters pathway

**Figure 8:**
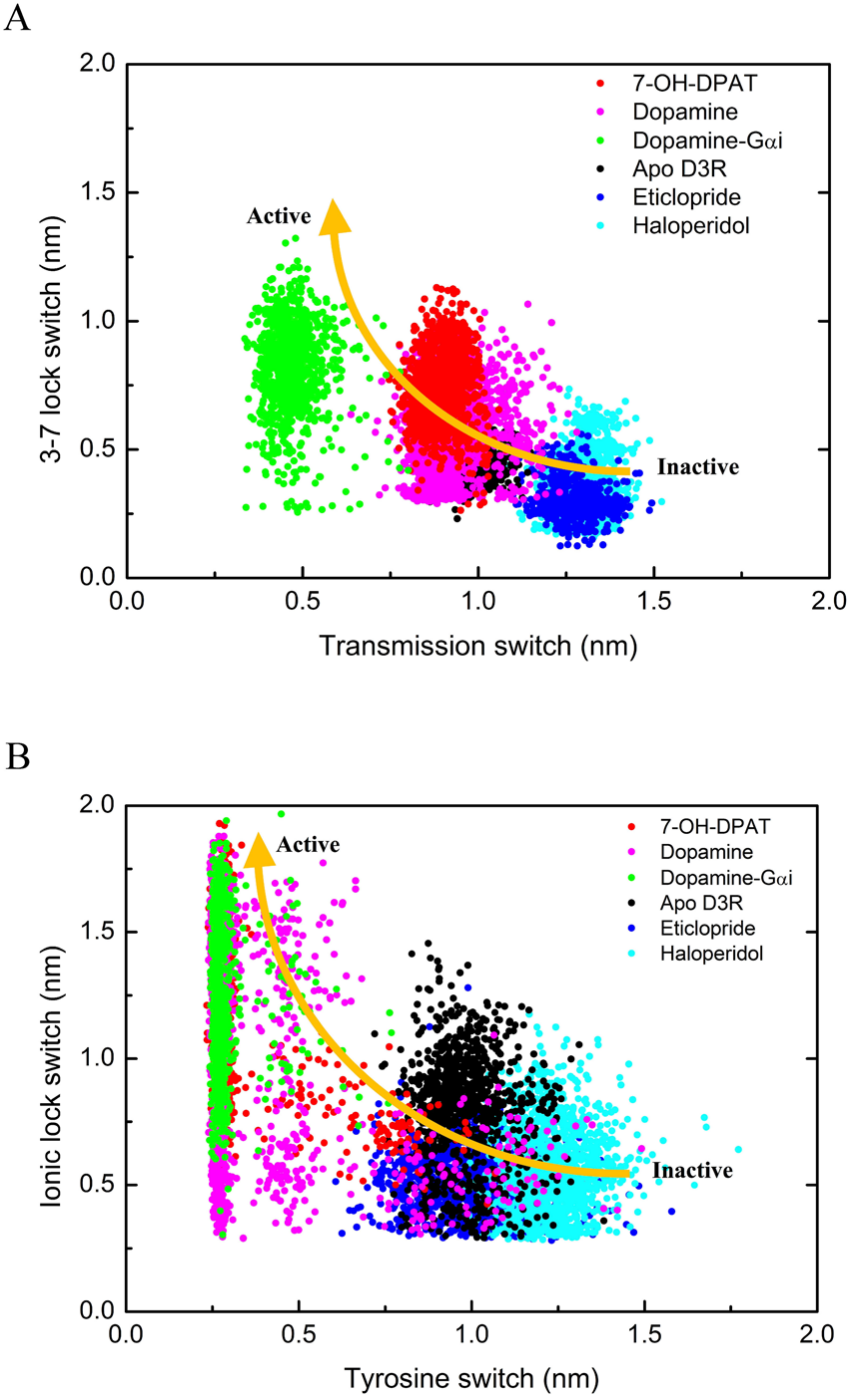
Scattering plots of distances between molecular switches for various D3R systems. (**A**) 3–7 lock and transmission switch distances, and (**B**) tyrosine toggle and ionic lock switch distances. Scatters represent snapshots sampled every 1 ns. From inactive state to active state, it is obvious to find that the 3–7 lock switch distance increases gradually to break the salt bridge and the transmission switch decreases with time. In D3R activation, the tyrosine toggle switch is decreasing whereas the ionic lock switch is increasing with time. Various drugs such as 7-OH-DPAT, dopamine, dopamine-G_αi_, apo, Eticlopride, and Haloperidol bound D3Rs are marked using red, pink, green, black, blue, and cyan circles, respectively.

GPCR activation occurs through a series of conformational changes or molecular switches. In D3R systems, the distances between four sets of molecular switch residues were plotted against time. The scattering of the 3–7 lock switch distance (D110^3.32^–Y373^7.43^) and transmission switch distance (W342^6.48^–P200^5.50^) was plotted for all systems (Fig. 8A). For (antagonist) eticlopride-bound and (inverse agonist) haloperidol-bound D3Rs, the distance of the 3–7 lock switch is approximately 0.35 nm, and the distance of the transmission switch distance is approximately 1.25 nm throughout the simulation. Both distances are consistent with the X-ray structure of eticlopride-bound D3R^17^ and other inactive GPCR systems such as rhodopsin^23^ and β_2_AR^34^. The maintained 3–7 lock switch and extensive transmission switch imply that the antagonist or inverse agonist-bound D3R is in its inactive state. By comparison, agonist-bound (7-OH-DPAT-, DA-, and DA-G_αi_-bound) D3Rs indicate that the distance of the 3–7 lock switch increased from 0.3 to 1.2 nm, and the distance of transmission switch was shorter than that of the eticlopride- and haloperidol-bound D3Rs (Fig. 8A). From the inactive to active state, the 3–7 lock switch distance increases gradually, as expected, to break the salt bridge, and the transmission switch distance decreases with time. The scattering of the tyrosine toggle switch distance (Y208^5.58^–Y383^7.53^) and the ionic lock switch distance (R128^3.50^–E324^6.30^) were also plotted for simulations in which various drugs were bound (Fig. 8B). For eticlopride- and haloperidol-bound D3Rs, the tyrosine toggle switches fluctuated near 0.8 and 1.25 nm during simulation, and the ionic lock switches fluctuated near 0.5 and 0.7 nm. For DA- and 7-OH-DPAT-bound D3Rs, the tyrosine toggle switches initially scattered and decreased from 1.2 to 0.26 nm, whereas the ionic lock switches increased from 0.3 to 1.8 nm with time. For DA-G_αi_-bound D3R, the tyrosine toggle switch was at approximately 0.3 nm and the ionic lock switch fluctuated from 0.6 to 1.8 nm. These molecular switches indicated that in D3R activation, the tyrosine toggle switch decreases whereas the ionic lock switch increases with time, which is consistent with previous studies on dopamine D3R^17^, adenosine A_2A_R^35^, and rhodopsin receptor^25^.

Continuous water pathways have been found in activated GPCRs^10,21,28,36,37^. Results of the present study are consistent with those of previous ones. For instance, continuous water pathways formed only upon DA-G_αi_-binding (active state), entry of water molecules was hindered by one hydrophobic layer HL2 near the cytoplasmic side when bound to DA and 7-OH-DPAT (intermediate state), and it was hindered by two hydrophobic layers when bound to haloperidol, eticlopride (inactive state), and apo D3R (Fig. 6). A comparison of the time marks of notable molecular switch movements and water density maps (Figs 6 and 8) revealed that the 3–7 lock switch restricts fewer water molecules from entering the intrareceptor region in eticlopride- and haloperidol-bound D3Rs, whereas the 3–7 lock switch disrupts the salt bridge to enable the entry of more water molecules entering in DA-, 7-OH-DPAT-, and DA-G_αi_-bound D3Rs. The distances of transmission and tyrosine toggle switches are larger in eticlopride- and haloperidol-bound D3Rs than that in DA-, 7-OH-DPAT-, and DA-G_αi_-bound D3Rs. The shorter distances of the transmission and tyrosine toggle switch residues may enable the two hydrophobic layers to prevent the passage of water molecules. The ionic lock switch is maintained to obstruct the passage of water molecules through the cytoplasmic side in eticlopride- and haloperidol-bound D3Rs, whereas the switch is disrupted to form hydrogen bond networks with water molecules for internal water molecules flowing into the cytoplasmic side in DA-, 7-OH-DPAT-, and DA-G_αi_-bound D3Rs.

### Proposed signal transduction pathway for D3R activation

**Figure 9:**
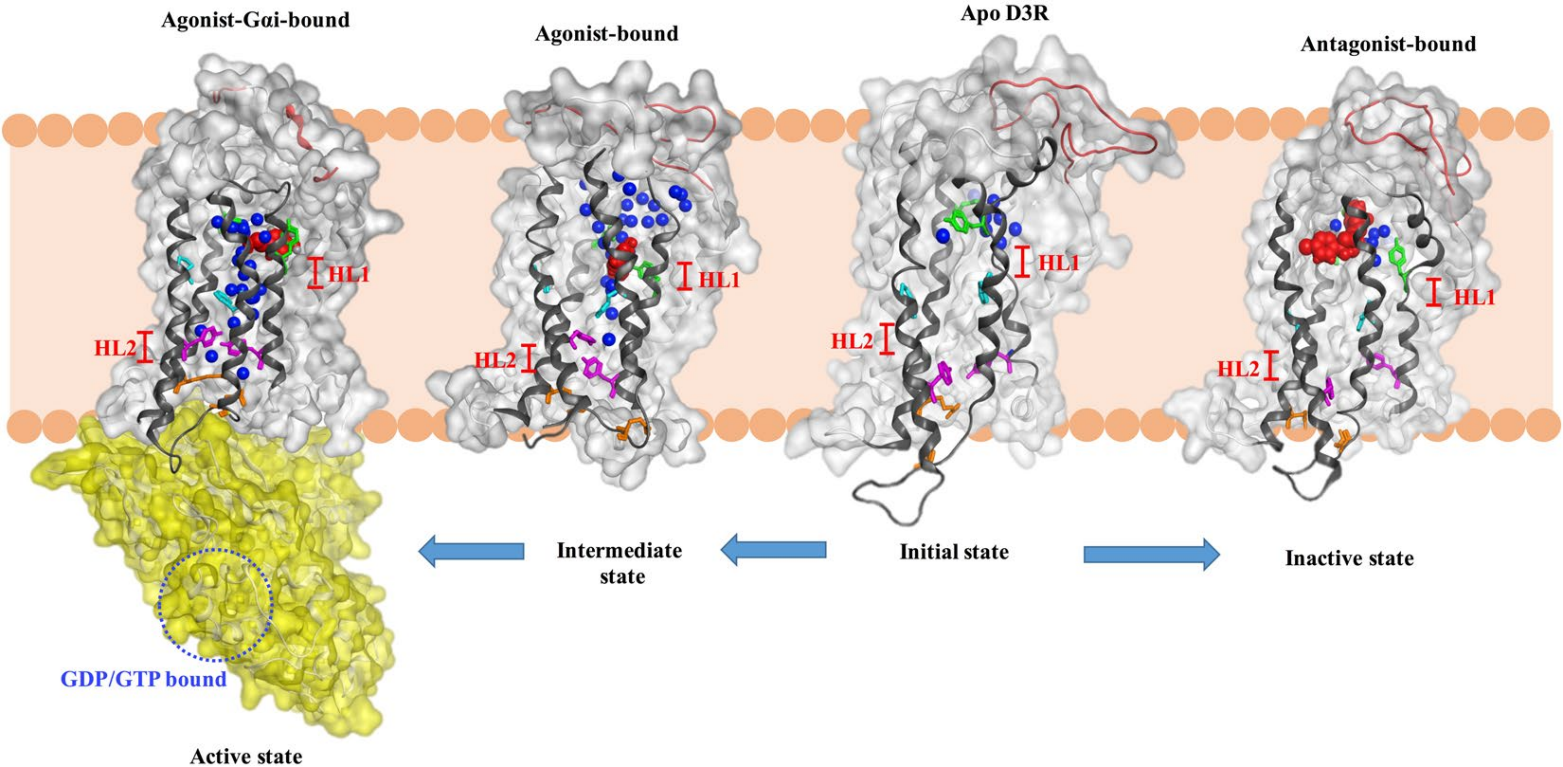
Complete activation mechanism of D3R from initial state to active state. The proposed complete activation mechanism correlates ligand-binding, conformational changes, internal water pathway and G protein binding. Waters are marked in blue balls; the side chains of molecular switch residues are marked in green (3–7 lock), cyan (transmission), purple (tyrosine toggle), and orange (ionic lock) sticks. The N-terminus is shown in red loop.

GPCRs have been considered molecular machines, whose activation and inactivation are conducted through the orchestration of molecular switches in the TM domain. These switches yield a high number of microstates and receptors bound to different ligands and occupy vastly different conformational spaces^38^. Although several studies have proposed the GPCR activation mechanism, which only focuses on molecular switches, the detailed mechanism correlating ligand binding, conformational changes, molecular switches, and internal water channels remains unclear. Based on our simulations, a complete activation mechanism is proposed for an agonist binding with D3R to recruit G protein association for signal transduction (Fig. 9). (i) As an agonist binds to D3R, the N-terminus may lie flat on top of binding cavity, forming a “lid-like” conformation to induce the ECL3 fluctuation for further molecular switches. (ii) The 3–7 lock switch is the first to be affected, and it disrupts the salt bridge to allow more water molecules to enter. Activating conformational changes have been characterized by movements within TMs 5, 6, and 7. (iii) TM6 rotates to approach TM5, which decreases the transmission switch distance and opens the hydrophobic layer (HL1) for allowing more water molecules to pass through. (vi) The water pathway shifts its direction and the tyrosine toggle switch also diminishes to break another hydrophobic layer (HL2) by relocation of Y208^5.58^ and Y383^7.53^ side chains, forming more hydrogen bond network with proximal water molecules. (v) The ionic lock switch further opens for water molecules flowing into the intracellular side, forming a hydrogen bond network with the DRY motif. (vi) In view of these events, the outward displacement of TM6 occurs to increase the volume of the G protein binding site for downstream signal transduction. The internal water molecules are hindered by the two hydrophobic layers (HL1 and HL2) at the initial or inactive state, and by only HL2 at the intermediate active state. (vii) Finally, the internal water molecules form a continuous water pathway in the active state, implying that HL2 is a determinant in the activation mechanism (Fig. 9). Previous studies showed conformational heterogeneity of β_2_AR from inactive state to active state may be important for engaging several alternative signaling and regulatory proteins^39,40^. In their studies, only the agonist bound to β_2_AR was in the intermediate state, whereas β_2_AR with agonist and nanobody 80 (mimic Gs protein) bound was in the active state^39^, and TM5 and TM6 showed the conformational changes during the activation process^40^, which are consistent with our porcupine analysis (Fig. 4) and activation model (Fig. 9). Previous MD simulations of A_2A_R, β_2_AR and P2Y_1_ receptor systems also showed that a hydrophobic layer next to NPxxY motif forms a gate as antagonist and only agonist bound to receptor, whereas the gate opens to form a continuous water channel under receptor activation, which is also similar to our activation model^21,41^.

## Conclusion

To our knowledge, the activation mechanism of D3R has yet to be elucidated. In the current study, MD simulations on a microsecond timescale were performed to elucidate this mechanism. The consistency of different N-terminus conformations could not be overlooked. Observation of the collective and correlated motions indicated that the N-terminal domain of D3R participates in receptor activation. Calculating the distances of molecular switch residues and G protein binding site volumes revealed that DA-G_αi_-bound D3R was most activated compared with the other systems. In addition, movements of TMs 5, 6, and 7 contributed significantly to receptor activation. A complete activation mechanism of D3R from the N-terminus to the cytoplasmic G protein binding site is proposed to indicate that the hydrophobic layer HL2 is dominant in the activation of D3R. In summary, this study clarifies how D3R may reach its active state conformation, and the findings could prove valuable in structure-based drug design for the treatment of CNS-related diseases.

## Materials and Methods

### Modeling of the N-terminus of D3R

While the D3R structure was crystallized (PDB code: 3PBL) by Stevens, R. C. *et al*.^17^ in 2010, the N-terminus of D3R is still lacking due to its highly flexible extracellular region. To investigate the role of N-terminal region in the binding and activation mechanism of D3R, the human neurokinin-1 receptor (NK_1_R) (PDB code: 2KSA, similarity: 32.3%) was therefore used as a template for homology modeling the N-terminus of D3R, created by using Phyre2 web server (http://www.sbg.bio.ic.ac.uk/phyre2/)^42^. The resolved structure of D3R with T4L removed was then connected with the homology modeled N-terminus using the Molecular Operating Environment software (http://www.chemcomp.com) (MOE2015.10). The constructed D3R with N-terminus was embedded into a POPC (16:0–18:1 diester PC, 1-palmitoyl-2-oleoyl-sn-glycero-3-phosphocholine) lipid bilayer for energy minimization and equilibration.

### Molecular docking of small compounds and G_αi_ subunit

Small compounds were manually built in the MOE software package (MOE2015.10). The topology and parameters files of small compounds, not supported in GROMACS program, were obtained from the GlycoBioChem PRODRG2 web server (http://davapc1.bioch.dundee.ac.uk/cgi-bin/prodrg) provided by Prof. Daan van Aalten^43^ under the GROMOS 53A6 force field which is also suitable for biomolecules. To validate the topology files generated by PRODRG2 program, we also use Automated Topology Builder and Repository web server (ATB ver. 2.2)^44^ to generate topology files for confirmation, which using a B3LYP/6-31 G* optimized geometry, GROMOS53A6 parameter set for bonded and van der Waals parameters, and RESP methods^45^ to generate charges and charge groups. The ATB 2.0 program has been validated by Mark, A. E. *et al*.^46^. To predict the preferable binding sites between D3R and small compounds, the MOE2015.10 software package was used to perform the molecular docking. In addition, the favorable binding sites between D3R and the G_αi_ subunit were predicted using the “*Dock Proteins*” protocol of Discovery Studio 3.5 (BIOVIA, http://accelrys.com).

### Molecular Dynamics (MD) simulations

After molecular docking, all preferable receptor-small compound complexes were inserted into a POPC lipid bilayer system (2 × 144 lipids) by removing overlapping lipids and water molecules for further MD simulations. Next, after energy minimization, solvated water boxes (9.6 nm × 9.6 nm × 9.6 nm) were added with ions (Na^+^ and Cl^−^) to generate 0.15 mol/L NaCl solution. All simulations were carried out with GROMACS-4.6.7 using GROMOS96 (ffG53a6) force field with an integration step size of 2 fs. Simulations were conducted in the *NPT* ensemble employing the velocity-rescaling thermostat at constant temperature 310 K, and 1 bar. Temperatures of the complexes, lipids and solvents were separately coupled with a coupling time of 0.1 ps. Semi-isotropic pressure coupling was applied with a coupling time of 0.1 ps and a compressibility of 4.5 × 10^−5^ bar^−1^ for the xy-plane as well as for the z-direction. Long-range electrostatics were calculated using the particle-mesh Ewald (PME) summation algorithm with grid dimensions of 0.12 nm and an interpolation order of 4. Lennard-Jones and short-range Coulomb interactions were cut off at 1.4 and 1.0 nm, respectively. Based on our previous study^33^ the protocol used for equilibration is as following: (i) temperatures were gradually increased from 100 K to 200 K and then to 310 K. Systems were run for 500 ps under each temperature. During these simulations, complex structures remained fully restrained (k = 1000 kJ mol^−1^ nm^−2^). (ii) At 310 K, restraints on the complex structure via the force constant k, were released in 3 steps. Starting from k = 500 kJ mol^−1^nm^−2^ to k = 250 kJ mol^−1^nm^−2^, and then to k = 100 kJ mol^−1^nm^−2^. Each step was run for 2.0 ns. After equilibration, production runs were carried out without any constraint on complex structures. Details for all simulations were listed in Table 2.

**Table 2:** Summary of simulation lengths and atom numbers.

Simulation system	Simulation length (μs)	Total number of atoms	Number of sodium ions	Number of chloride ions	Number of lipids
7-OH-DPAT	1.5	62080	80	88	238
DA	1.7	62682	80	88	238
DA-G_αi_	0.8^*^	64468	80	89	238
Apo D3R	1.0	61726	80	87	238
Eticlopride	1.5	61754	80	88	238
Haloperidol	1.5	61958	80	88	238

*Since the G_αi_ subunit is bound to the 1.0 μs D3R, the Dopamine-G_αi_-bound simulation is defined to start from 1 μs.

### Analyses of MD simulations

#### Volume calculations

The PyMol plugin KVFinder^47^, is a tool for cavity prospection and spatial characterization. This tool uses a geometrical grid-based method, in which a target protein is inserted into a 3D grid. Points within this grid are either occupied by the protein when lying inside the van der Waals (VDW) radii of protein atoms, or empty. Two probes, a larger one (*Probe Out*) and a smaller one (*Probe In*), are used to screen empty points. Each probe defines a surface that either overlaps or does not overlap grid points. Overlapped points must be inside the probe radius and away from the VDW radii of the protein. Step sizes were set to 0.6 Å, the *Probe In* size was set to 1.0 Å and the *Probe Out* size was set to 5.0 Å.

#### Generalized Cross Correlations and Principal Component Analysis

Using the generalized correlation analysis approach developed by Lange and Grubmüller^48^, correlated motions between C_α_ atoms in D3R residues were calculated based on mutual information (MI) with the *g_correlation* module of the GROMACS-3.3.3 package. Principal component analysis (PCA) reduces the dimensionality of an analyzed data set based on the covariance matrix calculated from atomic displacements throughout the trajectory. As a result, PCA extracts principal components (PCs) or eigenvectors that contribute the most to atomic displacements. PCA was carried out using the programs *g_covar* and *g_anaeig* from the GROMACS-4.6.7 package.

## Electronic supplementary material

Supplementary information

## References

[1] Missale C, Nash SR, Robinson SW, Jaber M, Caron MG (1998). Dopamine receptors: from structure to function. Physiol. Rev..

[2] Beaulieu JM, Gainetdinov RR (2011). The physiology, signaling, and pharmacology of dopamine receptors. Pharmacol. Rev..

[3] Lee T, Seeman P, Rajput A, Farley LJ, Hornykiewicz O (1978). Receptor basis for dopaminergic supersensitivity in Parkinson’s disease. Nature.

[4] Seeman P (2013). Schizophrenia and dopamine receptors. Eur. Neuropsychopharmacol..

[5] Wu J, Xiao H, Sun H, Zou L, Zhu L-Q (2012). Role of dopamine receptors in ADHD: a systematic meta-analysis. Mol. Neurobiol..

[6] Robbins TW, Everitt BJ (1999). Drug addiction: bad habits add up. Nature.

[7] Pescosolido N, Parisi F, Russo P, Buomprisco G, Nebbioso M (2013). Role of Dopaminergic Receptors in Glaucomatous Disease Modulation. Biomed Res Int.

[8] Trzaskowski B (2012). Action of Molecular Switches in GPCRs - Theoretical and Experimental Studies. Curr. Med. Chem..

[9] Warne T (2011). The structural basis for agonist and partial agonist action on a β1-adrenergic receptor. Nature.

[10] Farzan M (1997). HIV-1 entry and macrophage inflammatory protein-1β-mediated signaling are independent functions of the chemokine receptor CCR5. J Biol Chem..

[11] Congreve M, Marshall F (2010). The impact of GPCR structures on pharmacology and structure‐based drug design. Br J Pharmacol.

[12] Tautermann CS, Seeliger D, Kriegl JM (2015). What can we learn from molecular dynamics simulations for GPCR drug design?. Comput. Struct. Biotechnol. J..

[13] Rajagopalan L, Rajarathnam K (2004). Ligand selectivity and affinity of chemokine receptor CXCR1 Role of N-terminal domain. J Biol Chem..

[14] Szpakowska M (2012). Function, diversity and therapeutic potential of the N-terminal domain of human chemokine receptors. Biochem Pharmacol.

[15] D’ror RO (2011). Activation mechanism of the β2-adrenergic receptor. Proc Natl Acad Sci USA.

[16] Miao Y, Nichols SE, Gasper PM, Metzger VT, McCammon JA (2013). Activation and dynamic network of the M2 muscarinic receptor. Proc Natl Acad Sci USA.

[17] Chien EY (2010). Structure of the Human Dopamine D3 Receptor in Complex with a D2/D3 Selective Antagonist. Science.

[18] Grossfield A, Pitman MC, Feller SE, Soubias O, Gawrisch K (2008). Internal hydration increases during activation of the G-protein-coupled receptor rhodopsin. J. Mol. Biol..

[19] Angel TE, Chance MR, Palczewski K (2009). Conserved waters mediate structural and functional activation of family A (rhodopsin-like) G protein-coupled receptors. Proc Natl Acad Sci USA.

[20] Angel TE, Gupta S, Jastrzebska B, Palczewski K, Chance MR (2009). Structural waters define a functional channel mediating activation of the GPCR, rhodopsin. Proc Natl Acad Sci USA.

[21] Yuan S, Filipek S, Palczewski K, Vogel H (2014). Activation of G-protein-coupled receptors correlates with the formation of a continuous internal water pathway. Nat. Commun..

[22] Yuan S, Hu Z, Filipek S, Vogel H (2015). W246^6.48^ opens a gate for a continuous intrinsic water pathway during activation of the adenosine A2A receptor. Angew. Chem. Int. Ed..

[23] Kim JM (2004). Structural origins of constitutive activation in rhodopsin: Role of the K296/E113 salt bridge. Proc Natl Acad Sci USA.

[24] Deupi X, Standfuss J (2011). Structural insights into agonist-induced activation of G-protein-coupled receptors. Curr Opin Struct Biol.

[25] Standfuss J (2011). The structural basis of agonist-induced activation in constitutively active rhodopsin. Nature.

[26] Palczewski K (2000). Crystal structure of rhodopsin: A G protein-coupled receptor. Science.

[27] Ahuja S, Smith SO (2009). Multiple switches in G protein-coupled receptor activation. Trends Pharmacol Sci.

[28] Lange OF, Grubmüller H (2008). Full correlation analysis of conformational protein dynamics. Proteins: Structure, Function, and Bioinformatics.

[29] Scheerer P (2008). Crystal structure of opsin in its G-protein-interacting conformation. Nature.

[30] Kruse AC (2013). Activation and allosteric modulation of a muscarinic acetylcholine receptor. Nature.

[31] Cherezov V (2007). High-resolution crystal structure of an engineered human β2-adrenergic G protein–coupled receptor. Science.

[32] Tikhonova IG, Selvam B, Ivetac A, Wereszczynski J, McCammon JA (2013). Simulations of biased agonists in the β2 adrenergic receptor with accelerated molecular dynamics. Biochemistry.

[33] Liou JW (2014). *In Silico* Analysis Reveals Sequential Interactions and Protein Conformational Changes During the Binding of Chemokine CXCL-8 to its Receptor CXCR1. PLoS One.

[34] Rasmussen SG (2011). Crystal structure of the beta2 adrenergic receptor-Gs protein complex. Nature.

[35] Lebon G (2011). Agonist-bound adenosine A2A receptor structures reveal common features of GPCR activation. Nature.

[36] Liu W (2012). Structural basis for allosteric regulation of GPCRs by sodium ions. Science.

[37] Caliman AD, Swift SE, Wang Y, Miao Y, McCammon JA (2015). Investigation of the conformational dynamics of the apo A2A adenosine receptor. Protein Sci..

[38] Lee Y, Choi S, Hyeon C (2015). Communication over the Network of Binary Switches Regulates the Activation of A 2A Adenosine Receptor. PLOS Comput Biol.

[39] Nygaard R (2013). The Dynamic Process of β2-Adrenergic Receptor Activation. Cell.

[40] Kohlhoff KJ (2014). Cloud-based simulations on Google Exacycle reveal ligand modulation of GPCR activation pathways. Nat Chem.

[41] Yuan S (2016). The molecular mechanism of P2Y_1_ receptor activation. Angew. Chem. Int. Ed..

[42] Kelley LA, Sternberg MJE (2009). Protein structure prediction on the Web: a case study using the Phyre server. Nat. Protoc..

[43] Schuttelkopf AW, van Aalten DMF (2004). PRODRG - a tool for high-throughput crystallography of protein-ligand complexes. Acta Crystallogr..

[44] Malde AK (2011). An Automated force field Topology Builder (ATB) and repository: version 1.0. J. Chem. Theory Comput..

[45] Bayly CI, Cieplak P, Cornel W, Kollman PA (1993). A well-behaved electrostatic potential based method using charge restraints for deriving atomic charges: the RESP model. J. Phys. Chem..

[46] Koziara KB, Stroet M, Malde AK, Mark AE (2014). Testing and validation of the Automated Topology Builder (ATB) version 2.0: prediction of hydration free enthalpies. J Comput Aided Mol Des.

[47] Oliveira SH (2014). KVFinder: steered identification of protein cavities as a PyMOL plugin. BMC bioinformatics.

[48] Lange OF, Grubmüller H (2006). Generalized correlation for biomolecular dynamics. Proteins: Structure, Function, and Bioinformatics.

