# Supplementary material for: Activation-Induced Conformational Changes of Dopamine D3 Receptor Promote the Formation of the Internal Water Channel
Source: Sci Rep. 2017 Oct 6;7:12792. doi: 10.1038/s41598-017-13155-z (PMC5630584; doi:10.1038/s41598-017-13155-z)
Supplement: Supplementary file 1 — Supplementary information [file 41598_2017_13155_MOESM1_ESM.pdf]

Supplementary information

Activation induced conformational changes of dopamine D3 receptor promote formation of the internal water channel

Wei-Hsiang Weng, Ya-Tzu Li and Hao-Jen Hsu*

Department of Life Sciences, Tzu Chi University, Hualien 97004, Taiwan

Table S1: Docking scores for 7-OH-DPAT bound to D3R.

Pose No.	S	rmsd_refine (Å)	E_conf	E_place	E_score1	E_refine	E_score2
			(kcal/mol)				
1	-6.1431	2.5231	-5.9745	-50.7167	-9.3270	-31.2633	-6.1431
2	-6.1226	2.5414	6.5000	-56.4873	-9.3295	-23.5358	-6.1226
3	-6.0965	1.1673	-0.8704	-58.0837	-9.5507	-21.4632	-6.0965
4	-6.0313	1.8089	-8.3362	-46.4188	-9.0884	-22.8830	-6.0313
5	-6.0245	2.7030	-0.0370	-55.3790	-9.2949	-26.4149	-6.0245
6	-5.9808	2.7939	-5.0271	-54.1544	-10.2910	-30.1909	-5.9808
7	-5.9773	1.1788	-6.7444	-54.0107	-10.0600	-22.2461	-5.9773
8	-5.9362	1.9125	-3.9768	-47.6801	-9.4602	-29.8300	-5.9362
9	-5.9099	0.7842	-10.7123	-58.5124	-10.5182	-27.4526	-5.9099
10	-5.9075	2.1655	-5.7146	-54.7193	-9.3492	-34.7017	-5.9075
11	-5.8809	2.8119	-6.9931	-47.9028	-10.0624	-21.3464	-5.8809
12	-5.8725	2.6470	-7.4581	-77.3859	-6.7398	-26.3722	-5.8725
13	-5.8568	2.3375	-14.1822	-56.7982	-10.1858	-29.2806	-5.8568
14	-5.8487	1.0771	9.4445	-60.4933	-9.0129	-24.0013	-5.8487
15	-5.8331	1.4356	-5.4583	-47.1321	-9.3457	-24.3052	-5.8331

Pose1 with the lowest docking score based on docking module of MOE program was selected for further MD simulation.

Table S2: Docking scores for dopamine bound to D3R.

Pose No.	S	rmsd_refine (Å)	E_conf	E_place	E_score1	E_refine	E_score2
			(kcal/mol)				
1	-5.8253	2.2927	-58.7552	-55.6070	-8.9679	-41.5610	-5.8253
2	-5.7979	3.5270	-58.9163	-64.0429	-9.8880	-40.7484	-5.7979
3	-5.7768	2.7395	-57.5142	-60.7654	-10.2688	-42.1322	-5.7768
4	-5.7170	2.2842	-59.1238	-52.4101	-8.9845	-41.9423	-5.7170
5	-5.6663	0.7110	-56.2512	-73.6768	-8.8724	-37.1237	-5.6663
6	-5.6002	2.6099	-57.8909	-55.1282	-8.7801	-38.9001	-5.6002
7	-5.5242	0.7980	-59.8375	-54.5177	-9.7457	-35.0391	-5.5242
8	-5.4925	1.2667	-56.7969	-51.6739	-9.7673	-37.6220	-5.4925
9	-5.4897	0.7452	-54.5937	-56.1533	-9.3159	-38.4813	-5.4897
10	-5.3824	2.3739	-58.9002	-51.3594	-8.8222	-36.1832	-5.3824
11	-5.3276	1.4777	-59.8798	-51.2998	-10.1158	-33.0695	-5.3276
12	-5.1450	2.1224	-62.5562	-51.0281	-8.7982	-32.0048	-5.1450
13	-5.1251	1.2318	-57.9444	-51.3112	-9.1541	-25.6492	-5.1251
14	-5.0902	2.6290	-59.3996	-56.9605	-9.3559	-24.1906	-5.0902
15	-5.0511	1.3651	-59.00	-67.4582	-9.3754	-23.2195	-5.0511

Pose1 with the lowest docking score based on docking module of MOE program was selected for further MD simulation.

Table S3: Docking scores for Haloperidol bound to D3R.

Pose No.	S	rmsd_refine	E_conf	E_place	E_score1	E_refine	E_score2
		(Å)	(kcal/mol)				
1	-8.0103	1.7607	-27.0573	-47.1225	-9.5069	-52.4297	-8.0103
2	-7.8293	1.4599	-29.2204	-77.0184	-9.9426	-50.5723	-7.8293
3	-7.6306	1.4227	-22.1311	-48.3813	-10.9749	-52.4071	-7.6306
4	-7.6258	1.9492	-33.8460	-69.5062	-9.3540	-39.0519	-7.6258
5	-7.6171	2.1097	-24.9860	-40.9897	-9.5653	-52.4794	-7.6171
6	-7.5394	1.5279	-26.4002	-70.7361	-9.5132	-47.2252	-7.5394
7	-7.5236	1.3785	-32.0455	-70.9187	-9.8952	-43.8523	-7.5236
8	-7.5221	2.5037	-26.2959	-72.6861	-9.5191	-41.6475	-7.5221
9	-7.4844	1.8434	-20.2000	-63.0141	-10.6530	-43.1249	-7.4844
10	-7.4480	1.9119	-27.4746	-43.7060	-11.4986	-37.6237	-7.4480
11	-7.4163	1.4290	-25.9291	-83.3997	-11.1195	-50.9418	-7.4163
12	-7.4039	1.9503	-32.5958	-55.0637	-9.3730	-44.1090	-7.4039
13	-7.3584	1.6445	-28.1842	-77.4255	-10.9493	-40.4337	-7.3584
14	-7.3079	2.7335	-26.2678	-72.7379	-10.1259	-40.6179	-7.3079
15	-7.2856	1.7901	-30.2688	-75.4933	-10.2248	-36.1302	-7.2856

Pose1 with the lowest docking score based on docking module of MOE program was selected for further MD simulation.

Table S4: TM scores of ligand-bound D3R complex systems

System	TM scores	
Dopamine-bound D3R	At 100 ns	At 200 ns
Replica_1	0.8474	0.8420
Replica_2	0.8149	0.7753
Replica_3	0.8116	0.7905

System	TM scores	
Haloperidol-bound D3R	At 100 ns	At 200 ns
Replica_1	0.8157	0.8056
Replica_2	0.7784	0.7875
Replica_3	0.8124	0.7581

TM-score is a metric for measuring the structural similarity of two protein models which are initial complex D3R and 100 ns complex D3R or 200 ns complex D3R. TM-score has the value between 0 and 1, where 1 indicates a perfect match between two structures.

A

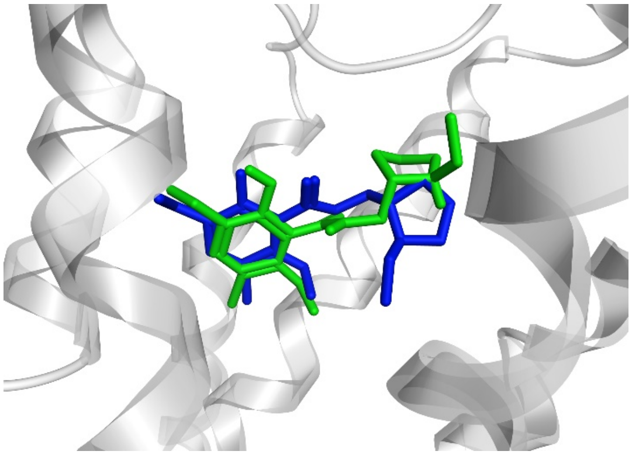

B

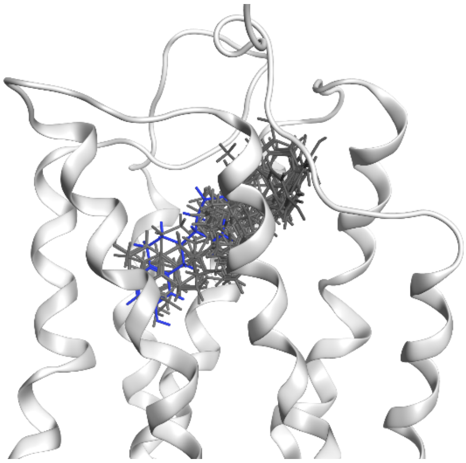

C

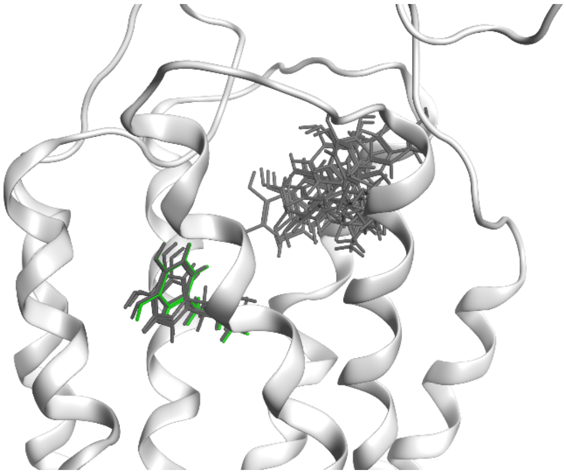

D

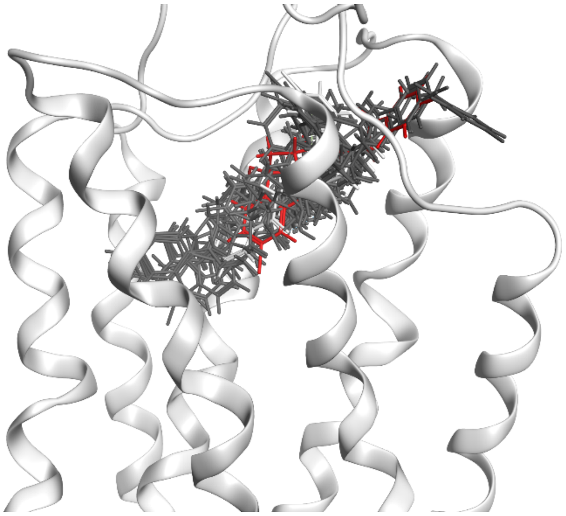

Figure S1: Molecular docking results for various small ligands binding to D3R. (A) Result of Eticlopride redocked to D3R. Original position in crystal structure (blue molecule) is quite similar to the redocked position (green molecule). (B) Superposition of molecular docking results of top 15 ranking poses for 7-OH-DPAT-bound D3R. Blue color pose is with the lowest docking score selected for MD simulations. (C) Superposition of molecular docking results of top 15 ranking poses for dopamine-bound D3R. Green color pose is with the lowest docking score selected for MD simulations. (D) Superposition of molecular docking results of top 15 ranking poses for haloperidol-bound D3R. Red color pose is with the lowest docking score selected for MD simulations.

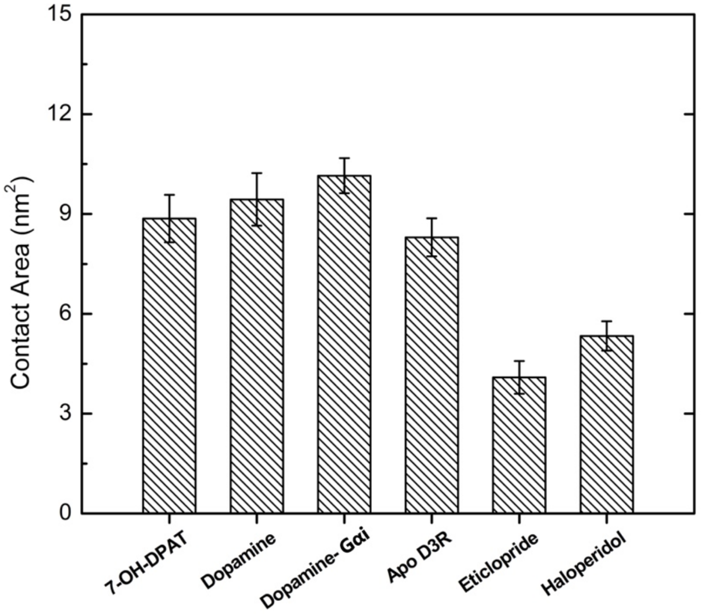

Figure S2: Contact areas between the N-terminus and transmembrane region. 7-OH-DPAT, dopamine, dopamine-G_{α} systems showed larger contact areas ranging from 8.0 to 10.5 nm^2, consistent with the 'lid-like' conformation of the N-terminus. The Eticlopride and Haloperidol-bound systems, on the other hand, showed smaller contact areas that ranged from 4.0 to 5.5 nm^2.

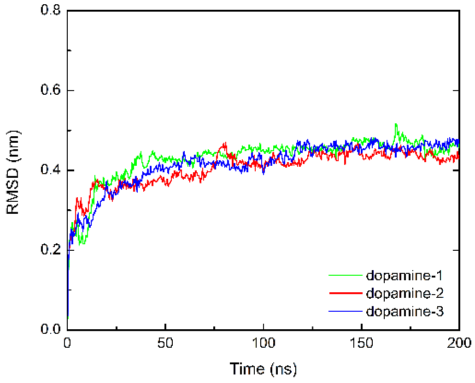
RMSD (nm)
Time (ns)
dopamine-1
dopamine-2
dopamine-3

A

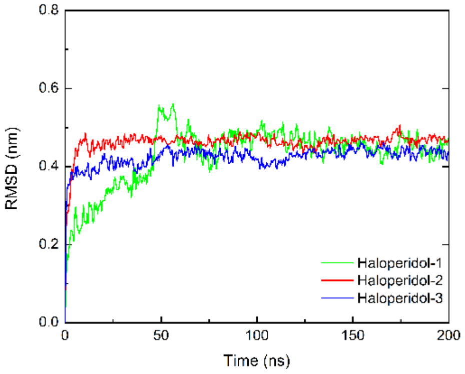
RMSD (nm)
Time (ns)
Haloperidol-1
Haloperidol-2
Haloperidol-3

B

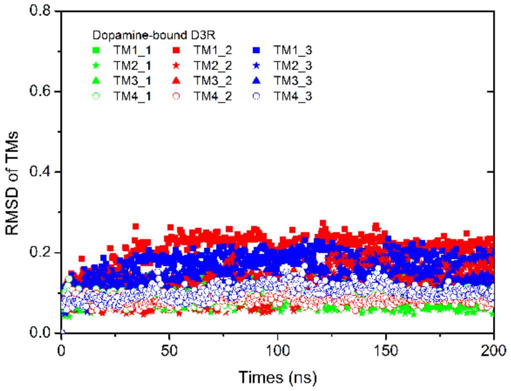
Dopamine-bound D3R
TM1_1 TM1_2 TM1_3
TM2_1 TM2_2 TM2_3
TM3_1 TM3_2 TM3_3
TM4_1 TM4_2 TM4_3
RMSD of TMs
Times (ns)

C

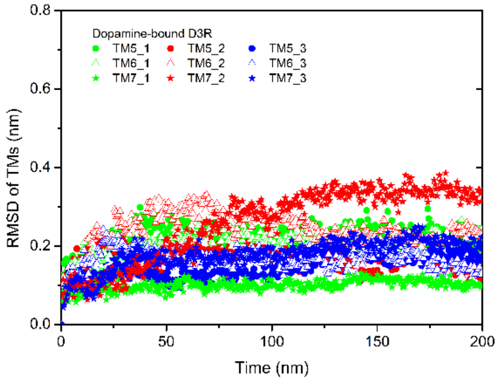
Dopamine-bound D3R
TM5_1 TM5_2 TM5_3
TM6_1 TM6_2 TM6_3
TM7_1 TM7_2 TM7_3
RMSD of TMs (nm)
Time (nm)

D

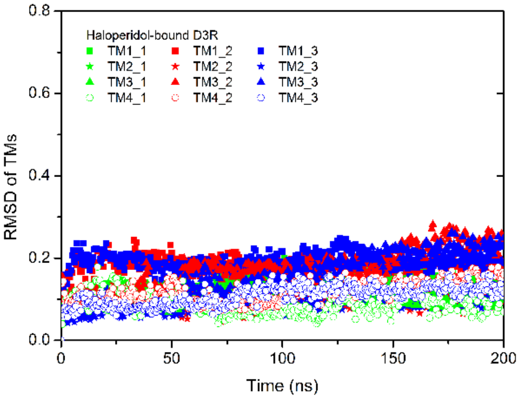
Haloperidol-bound D3R
TM1_1 TM1_2 TM1_3
TM2_1 TM2_2 TM2_3
TM3_1 TM3_2 TM3_3
TM4_1 TM4_2 TM4_3
RMSD of TMs
Time (ns)

E

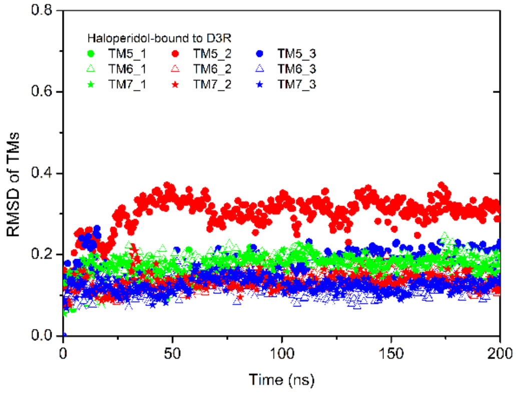
Haloperidol-bound to D3R
TM5_1 TM5_2 TM5_3
TM6_1 TM6_2 TM6_3
TM7_1 TM7_2 TM7_3
RMSD of TMs
Time (ns)

F

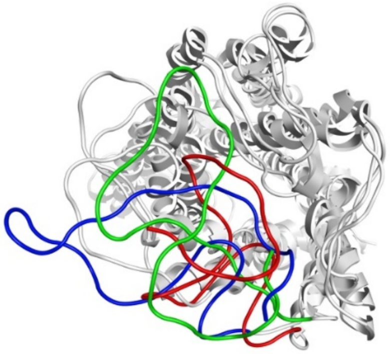

G

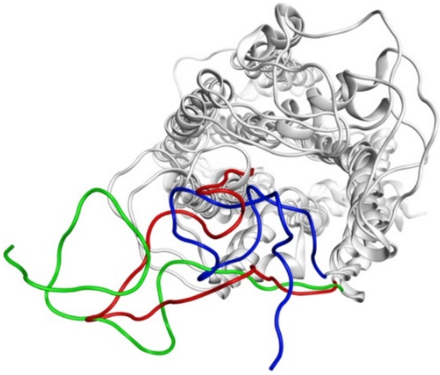

H

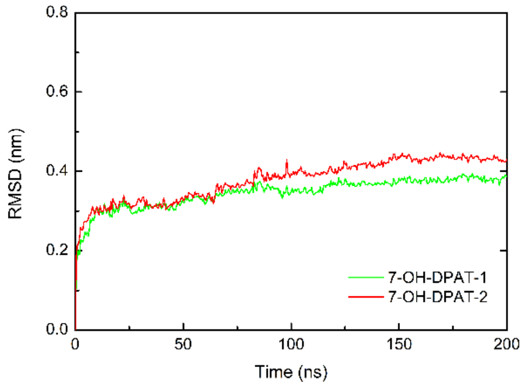
0.8
0.6
0.4
0.2
0.0
RMSD (nm)
0
50
100
150
200
Time (ns)
7-OH-DPAT-1
7-OH-DPAT-2

I

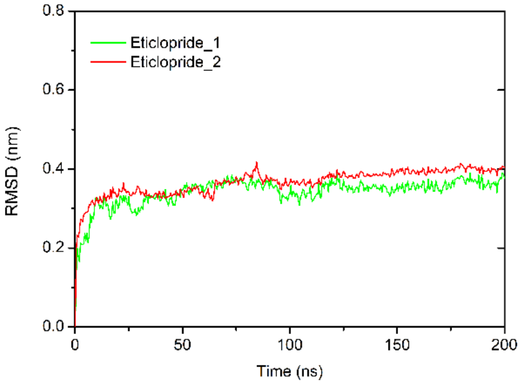
0.8
0.6
0.4
0.2
0.0
RMSD (nm)
0
50
100
150
200
Time (ns)
Eticlopride_1
Eticlopride_2

J

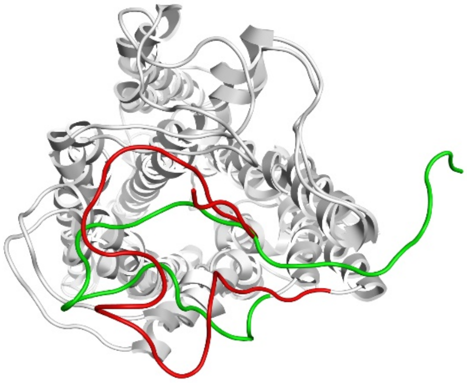

K

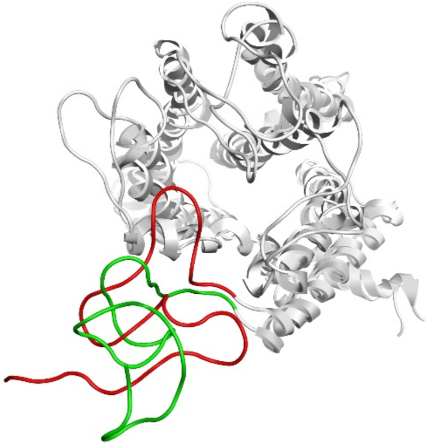

L

Figure S3: Comparison of original and repeated systems. Root mean square deviations (RMSDs) of (A) Dopamine-, (B) Haloperidol-, (I) 7-OH-DPAT- and (J) Eticlopride-bound D3R backbone atoms. (C) TM1~4 of dopamine- (D) TM1~4 of Haloperidol- (E) TM5~7 of dopamine- (F) TM5~7 of Haloperidol-bound D3R systems. The superposition of N-terminus conformations of (G) Dopamine-, (H) Haloperidol-, (K) 7-OH-DPAT-, and (L) Eticlopride-bound D3Rs.

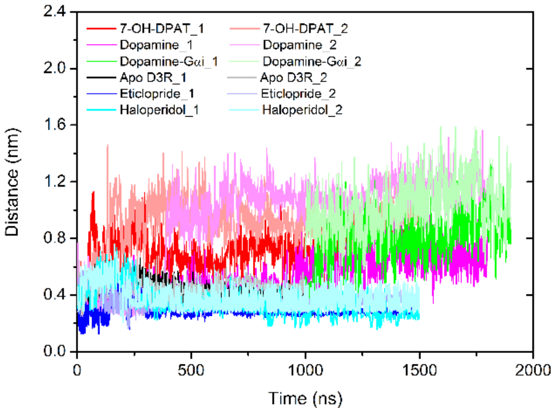

A

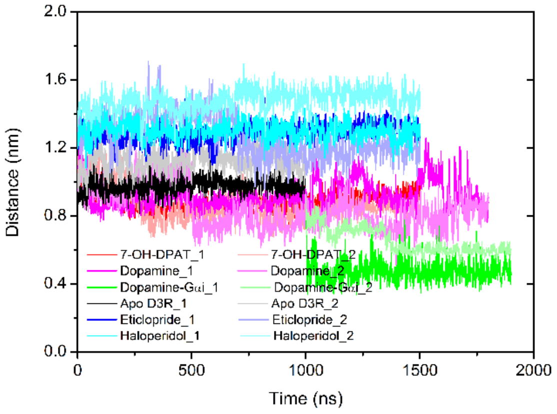

B

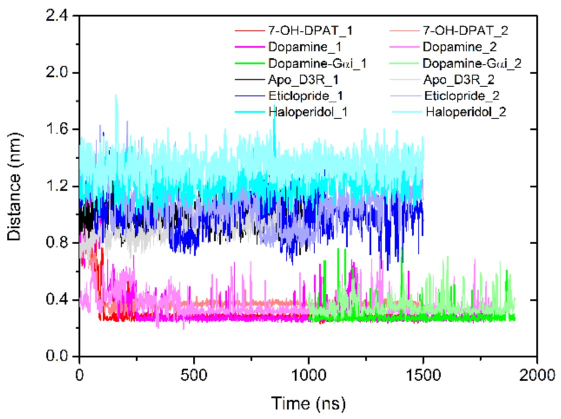

C

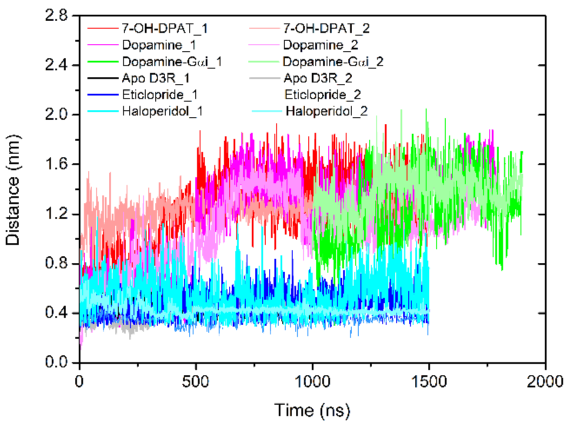

D

Figure S4: Measured distances between various molecular switches of the replicate simulations for each system. Each system was repeated two times under the same simulation conditions but with different initial velocity. Dark color is for first time, and light color is for second time. (A) 3-7 lock switch residues, the oxygen atoms of side chains of D110$^{3.32}$ and Y373$^{7.43}$ (B) Transmission switch, the center of masses (COMs) of W342$^{6.48}$ and P200$^{5.50}$ side chains (C) Tyrosine toggle switch, the oxygen atoms of side chains of Y208$^{5.58}$ and Y383$^{7.53}$ (D) Ionic lock switch, the nitrogen atom of R128$^{3.50}$ and the oxygen atom of E324$^{6.30}$. 7-OH-DPAT-, dopamine-, dopamine-Gαi, apo D3R, eticlopride-, and haloperidol-bound D3Rs are marked using red, pink, green, black, blue, and cyan lines, respectively.
